# Ensuring Safe Continuity of Care: Emergency Psychiatry Presentations and Communication with Primary Care– A Quality Improvement Project

**DOI:** 10.1192/bjo.2026.11378

**Published:** 2026-06-30

**Authors:** Sarah Fitzgerald, Rebecca Conlan-Trant, Ana-Maria Clarke

**Affiliations:** Mater Misericordiae University Hospital, Dublin, Ireland

## Abstract

**Aims:**

Patients presenting to the Emergency Department (ED) with acute mental health concerns are referred to liaison/on-call psychiatry for assessment. A proportion of these patients leave ED before psychiatric review takes place. The HSE and National Clinical Programme for Self-Harm and Suicide-related Ideation (NCP-SHSI) emphasise that clear documentation and timely communication with primary care are essential to ensure continuity of care, even when psychiatric assessment is not completed. Given the clinical risks associated with acute mental health presentations, ensuring that GPs are informed when a patient leaves prior to psychiatric assessment is crucial for patient safety, follow-up planning and clinical governance.

Aimswere:

To evaluate compliance with the HSE and NCP-SHSI regarding GP communication for patients who leave ED prior to psychiatric assessment.

To analyse risks associated with lack of GP communication in this patient cohort.

To implement a risk treatment plan.

To re-evaluate compliance following implementation of the risk treatment plan.

**Methods:**

All referrals to liaison/on-call psychiatry are recorded on the Siilo app. All referrals over a one month period were reviewed. Patients who left ED before psychiatric assessment were identified. Electronic patient records were reviewed to determine whether GP correspondence had been sent.

Risk identification, description and analysis were undertaken as per the HSE Enterprise Risk Management Policy and Procedures 2023.

Following implementation of a risk treatment plan, all referrals from ED over a two week period were reviewed.

**Results:**

A total of 167 psychiatry referrals from the ED were initially reviewed. Twenty patients left ED prior to psychiatric assessment. GP letters were sent for only 20% of these patients, compared with 57.8% of patients who completed assessment. Overall, GP correspondence was sent for 53.3% of ED presentations. When analysis was limited to patients with a registered GP, correspondence was sent in 60.1% of cases, leaving 39.9% without documented GP communication despite eligibility.

Using the HSE impact, likelihood, risk scoring and rating matrices, this lack of communication was identified as a medium risk.

**Conclusion:**

Initial findings demonstrated a significant shortfall in GP correspondence, highlighting an area for quality improvement in continuity of care and communication with primary care services.

A risk treatment plan was created, involving education sessions for the liaison psychiatry NCHDs, CNSs and all psychiatry NCHDs on the local on-call rota.

Re-evaluation after implementation of risk treatment plan demonstrated significantly improved compliance. Ongoing monitoring is recommended to ensure the sustainability of quality improvement, particularly following periods of high staff turnover.

